# Multi-layer modelling of masonry structures strengthened through textile-reinforced mortar

**DOI:** 10.12688/openreseurope.15233.2

**Published:** 2023-02-21

**Authors:** Ingrid Boem

**Affiliations:** 1 Department of Concrete and Masonry Structures, Czech Technical University in Prague, Praha 6, 16629, Czech Republic

**Keywords:** Seismic protection, Masonry strengthening, Composites, TRM, CRM, Numerical modelling, OOFEM

## Abstract

Background

Textile-reinforced mortar (TRM) is an innovative strategy for the reduction of the seismic vulnerability of existing masonry buildings consisting in the application on the masonry surface, of a mortar coating with fiber-based grids or textiles embedded. The paper presents the calibration and application of a simplified modelling approach, based on multi-layered elements, for the simulation of existing masonry elements and structures strengthened through TRM.

Methods

The strengthened masonry is modelled by using 20-nodes brick elements formed by a stacking sequence of layers representing the different material components (the masonry, the mortar coating and the embedded reinforcement). The nonlinear behavior of the materials is considered and calibrated on the basis of experimental characterization tests on individual components available in the literature. The simplified assumption of perfect bond among layers is considered.

Results

Non-linear static analyses are performed on samples of increasing complexity: elementary panels, structural elements (piers and spandrels) and a pilot building. The results of some tests on TRM strengthened masonry, available in the literature, are considered to assess the model reliability in terms of capacity curves and collapse mode. The model is capable of detecting the typical failure mechanism of both unstrengthened and TRM strengthened masonry, namely the diagonal cracking, the in-plane bending and the out-of-plane bending and is able to detect the activation also of mixed failure modes, that often occur in actual configurations.

Conclusions

Given the coarse mesh size and the smear plasticization assumption, the model is not suitable for the rigorous reproduction of individual cracks but represents a good compromise between the goal to grasp the structural performances at the wide scale, including failure modes, and the analysis optimization.

## Introduction

The European and worldwide building heritage consists of many historic masonry buildings that often need retrofitting interventions, due to structural deficiencies related to durability, modifications, fatigue, cyclic stresses, accidental actions. In particular, the experience has shown that these massive structures, traditionally conceived to withstand vertical loads, are particularly vulnerable to seismic actions ^1, 2^. This vulnerability is typically influenced by the structural consistency (connections among the walls and between walls and floors), the distribution of the resistant elements and the masonry integrity and resistance. On this latter, the main deficiencies are generally related to the poor tensile resistance of masonry, despite its good compressive strength. Thus, the introduction of tensile resistant elements can mitigate the seismic damage in masonry structures.

In this context, within the last 20 years, innovative strengthening systems for the seismic protection of existing masonry buildings, the textile reinforced mortars (TRMs), have gradually spread in the refurbishment sector ^3^. The technique consists of the application, on the masonry surfaces, of a mortar coating with a fiber-based reinforcement layer embedded. These systems are particularly suitable for application on existing masonry, since they combine the use of a high tensile resistant, un-corrosive material (the fiber-based reinforcement) with an inorganic matrix (the mortar), which is easy to apply on rough surfaces such as masonry and can assure mechanical and chemical compatibility with the substrate and provide fire and UV-ray protection to the fibers ^4^. Several material combinations have been proposed for TRM, differing for the nature and format of the reinforcement ( *e.g.* glass, carbon, basalt, in the form of textiles or meshes) and the type and thickness of mortar matrix.

The development of these modern techniques has actively involved the field of scientific research at different levels: the study of the mechanical and chemical behaviour of the compounds ^5– 10^, the testing of TRM strengthened masonry elements ^11– 16^, the calibration of numerical and analytical methods for the performance prediction ^17– 21^. The main research achievements in these fields have been recently reviewed, analysed and discussed by the author ^22, 23^: it emerged a populated and variegated experimental scenario. Clearly, as the scale and the complexity of the tests increases, the number reduces, due to the higher experimental effort; moreover, the great variability in the materials combinations, geometry, loading and boundary conditions, makes unable to cover experimentally all possible arrangements. Numerical models can overcome these intrinsic limits of the experimental tests, allowing to investigate on a wider number and more complex configurations. But most of numerical studies available are currently limited to the reproduction of laboratory tests on elementary specimens or, in a few cases, on the structural elements ( *i.e.* a pier or a spandrel). This is due, on one side, to the very limited number of tests available for validation at the large scale and, on the other, to the high computational effort of most of the available numerical models, unsuitable for an efficient application at the large scale, as observed by Oliveira *et al.* ^24^. Moreover, the analysis of the available numerical studies points out the lack of a comprehensive approach, rather than models calibrated and applied for the reproduction of a specific test setup and combination of materials.

The purpose to perform simplified, time-efficient reliable simulations for TRM strengthened structures led some researchers to switch from a detailed, micro-modelling approach, based on the modelling of individual components and interfaces, to a smeared, macro-modelling approach, by combining a series of different layers under perfect bond assumption. For example, Wang *et al.* ^25^, using software DIANA, coupled plies of quadrilateral 8-noded shell elements (mesh size 5 mm) representing the masonry and the mortar matrix, the latter provided also with an embedded reinforcement grid. The model, calibrated on the basis of experimental characterization tests on TRM coupons, was applied to the pushover analysis of a masonry façade with openings (governed by flexural response), to evaluate the benefits of TRM. To properly face also with out-of-plane loading conditions, the model was improved by Oliveira *et al.* ^24^, by using layered shell elements instead of coupling simple shell elements; it was applied to the simulation of a masonry C-shape assemblage with a central opening, strengthened with TRM at one or both sides. The model of Noor-E-Khuda *et al.* ^26^, developed in ABAQUS environment, was based on layered shells (mesh size 100 mm), composed of an inner layer, representing the masonry, and the outer layers, for the equivalent TRM material. An experimental-numerical comparison was made when simulating the out-of-plane, non-linear analysis of solid masonry panels made of dry-stack concrete blocks and strengthened with TRM. Ivorra *et al.* ^27^ used non-linear shell-layered elements (mesh size 100 mm) to model, with SAP2000, solid brick walls with and without opening, loaded in-plane (shear-compression). In particular, for the masonry, two overlapping layers accounted one for the mechanical characteristics in compression/tension and one for those in shear; an additional layer, representing TRM as an equivalent material, was applied at the two faces. All these analyzes provided preliminary indications reasonably realistic but lack real, large-scale validation of the results through comparison with experimental tests.

**Figure 1.**
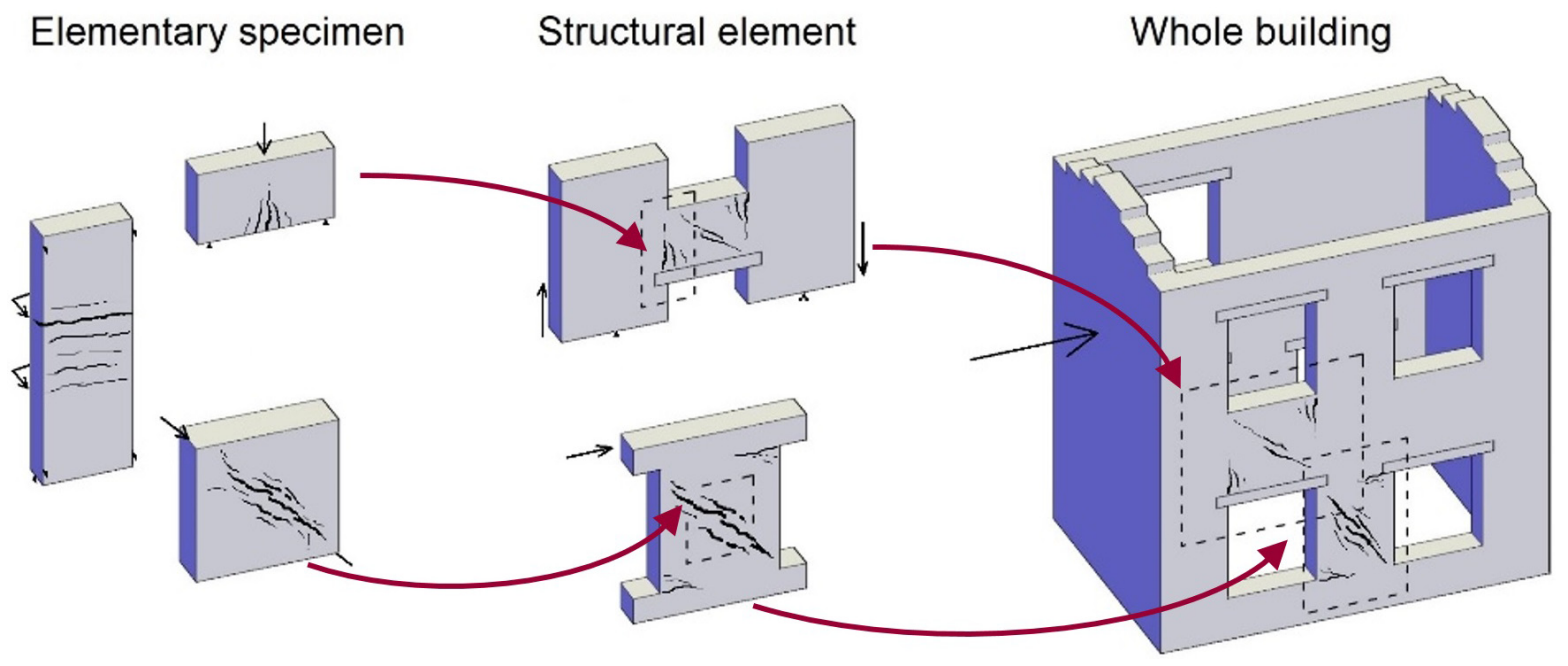
Schematization of the three scale application of the multi-layer modelling (MLM).

This paper deals with the scopes of the EU-funded “ conFiRMa” project -, aimed at the development of numerical methods for the study on the structural performances of TRM strengthened masonry by means of nonlinear static analysis. Different modeling strategies were calibrated, varying the scale of investigation: detailed modelling of components and interfaces, to investigate at the small scale (TRM coupons and elementary masonry samples strengthened with TRM); intermediate multi-layer modelling, for investigating at the medium and large scale level (structural elements, walls and buildings, strengthened with TRM); simplified equivalent frame modelling with lumped plasticity, to perform global analysis (large, building scale). In fact, widening the scale level, simplification and optimization procedures are necessary to obtain computationally efficient models, but the reliability has to be preserved. The detailed level modelling (DLM) has already been calibrated, validated and applied for sensitivity analysis of TRM coupons and TRM strengthened elementary masonry elements, as documented in 22, 28. As continuation, this paper focuses on the intermediate, multi-layer modelling (MLM) approach, presenting a simple but reliable way to evaluate the effects of TRM on masonry structures, taking into account the typical failure modes. At first, the characteristics of the strengthening technique and of the numerical MLM features are described. Then, the MLM is applied to the simulation of TRM strengthened masonry samples at three different scale levels ( Figure 1): elementary specimen, structural element and building. To prove the reliability of the numerical models, comparison is made with the results of experimental tests. It is specified that all the experimental test results mentioned in this paper refer to previous studies, available in the literature; this paper consists only in numerical simulations with the MLM. In the prospect of an open science approach, the finite element code adopted and the input files of the models herein described are available for free consultation and use ^29, 30^.

## Strengthening with CRM

The composite reinforced mortar (CRM) technique herein investigated identifies a type of TRM strengthening system consisting in the application, on the masonry wall surfaces, of a mortar layer having a nominal, minimum thickness of 30 mm, with alkali-resistant glass fiber-reinforced polymer (GFRP) pre-formed grids embedded. Typically, the GFRP grid has a squared grid pitch of 66x66 mm ^2^, with a cross section of dry fibers equal to 3.8 mm ^2^ in each yarn (id. “66S”). The grid is produced by twisting the yarns in the warp direction and weaving them across those in the weft direction, which fibers remain parallel.

**Figure 2.**
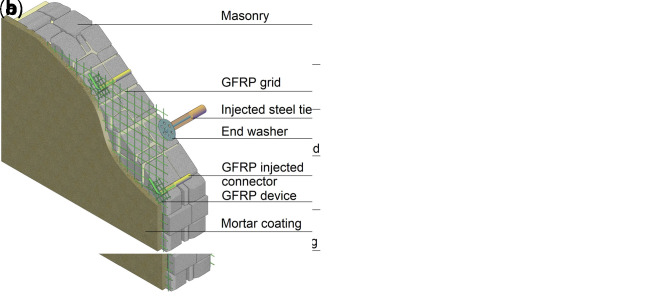
The composite reinforced mortar (CRM) strengthening technique: application on ( **a**) both sides or ( **b**) one side only. Acronym GFRP stands for glass fiber-reinforced polymer.

CRM is usually applied on both sides of the masonry walls, but also one-side application is possible. In the former case ( Figure 2a), the reinforced mortar coating is combined with the introduction of couples of GFRP L-shape passing-through connectors (6/m ^2^), injected into holes drilled in the masonry and provided with GFRP grid devices, to improve collaboration with the substrate. In the latter ( Figure 2b), GFRP connectors applied on the strengthened side (4/m ^2^) are combined with artificial diatones (2/m ^2^), introduced to contrast possible leafs separation in case of multiple-wythe masonry. Such diatones can be made *e.g.*, of stainless steel threaded rods inserted in fabric sleeves injected with high-performance grout and provided with specific end washers.

## Main features and calibration of the numerical model

The code OOFEM was adopted for the simulations: it is a free, open source code for finite element modelling (FEM) with object oriented architecture for solving mechanical, transport and fluid mechanics problems ^31, 32^. It is released under GNU Lesser General Public License (LGPL v2.1) and provides modular and extensible environment. The current version is OOFEM 2.5 ^29^. Detailed information about the type of elements and materials used in the simulations, mentioned in the following, can be found in the OOFEM manuals section, available online.

**Figure 3.**
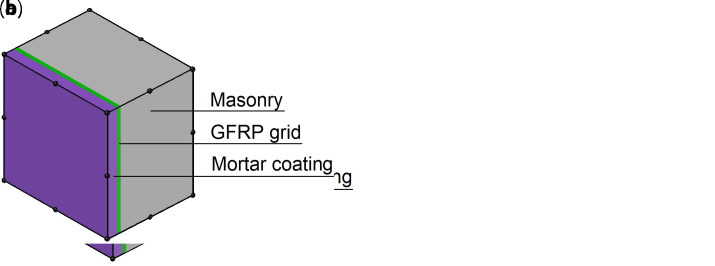
Layered element for the multi-layer modelling (MLM): composite reinforced mortar (CRM) applied on ( **a**) both sides or ( **b**) one side only. Acronym GFRP stands for glass fiber-reinforced polymer.

The models are composed of 20-nodes brick elements ( *QSpace*), with dimensions 167x167x *t* mm ^3^ (being *t* the overall wall thickness). The mesh dimension was chosen so that the aspect ratio (ratio between largest and smallest characteristic dimension) was maintained in the range 1.5-3, considering the typical thickness of existing masonry walls. This was find, through a preliminary model sensitivity analysis, a good compromise to ensure the accuracy of the representation and facilitate the convergence. The elements have a layered cross sections ( *LayeredCS*): a sequence of different plies arranged along the sample thickness are defined, representing the masonry, the fiber-based reinforcement and the mortar coating ( Figure 3). The layered elements are based on the simplified hypothesis that the layers are perfectly bonded to each other and that cross sections remain planar after deformation. Different thickness and material characteristics for each layer can be specified; the position of mid-surface is located by default at average thickness position or can be set manually (normal and bending forces are then computed with its regards). The Gauss integration rule is used for setting up integration points through the thickness of each layer and it is possible to indicate different number of integration points per individual layer. Specifically, 6 Gauss points were set for the masonry layer, 3 for the mortar coating and 1 for the GFRP layer.

Nonlinear-static analyses at displacement control were performed (Newton-Rapshon solver, with relative displacement and force convergence norms set to 3·10 ^-3^) by considering materials nonlinearities. In the way of simplification, all the materials were assumed homogeneous and isotropic; the definition of the material properties came from experimental basis.

A unitary thickness was assigned to the layer representing the GFRP grid; the material parameters were set on the basis of available experimental tensile tests on yarns, but the actual properties were scaled, so to be smeared over the uniform layer ( Table 1). The material behavior ( *Idm1*) was assumed to be linear elastic in tension, until reaching the peak deformation: the mean values of both resistance and ultimate strain in the two orthogonal main directions of the grid were considered. A linear decay of resistance was then assumed, rather than brutally brittle; in fact, although the single yarn approximately showed an elastic-brittle behavior, the "group effect" given by the progressive breakage of concurrently stressed yarns typically determined a slightly gradual softening.

**Table 1.** Main calibrated numerical parameters adopted for the GFRP layer.

GFRP grid	66S	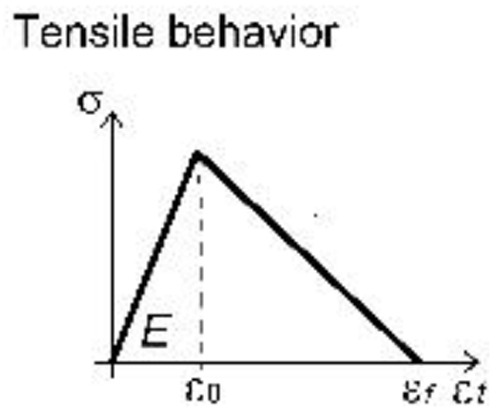
OOFEM material type	*Idm1*	
Young modulus *E*	3.81 GPa	
Poisson modulus *n*	0.01	
Comp. strength *f _c_*	-	
OOFEM Equiv. strain type	modified Mises	
Damage law	linear	
Peak strain *ε*_0_	1.8%	
Ultimate strain *ε _f_*	6.0%	
For unspecified parameters, the OOFEM default values are used - http://www.oofem.org/doku.php?id=en:manual		

A concrete-damage plasticity material model ( *Cdpm2* - 33) was considered for both the masonry and the mortar of the coating, also in accordance with the assumptions of the DLM previously calibrated ^22, 28^: possible materials failures for both cracking in tension and crushing in compression were, thus, considered.

Generally, the parameters of the mortar coating ( Table 2) can be set on experimental basis (characterization tests on cylinders/prisms) or, when lacking, on the values reported in the technical sheets provided by the mortar producers. However, particular attention has to be addressed to the calibration of the mortar post-cracking behavior. Practically, due to the smear-crack approach of the MLM, the mortar fracture energy needs to be fictitiously increased so to take into account for the tension stiffening effect of the mortar between cracks. In such a way, the combined effect of the plaster layer and the GFRP layer can macroscopically reproduce the typical trilinear behavior in tension of the CRM composite material, broadly analyzed and discussed when applying the DLM approach ^22^.

**Table 2.** Main calibrated numerical parameters adopted for the mortar.

MORTAR	C8	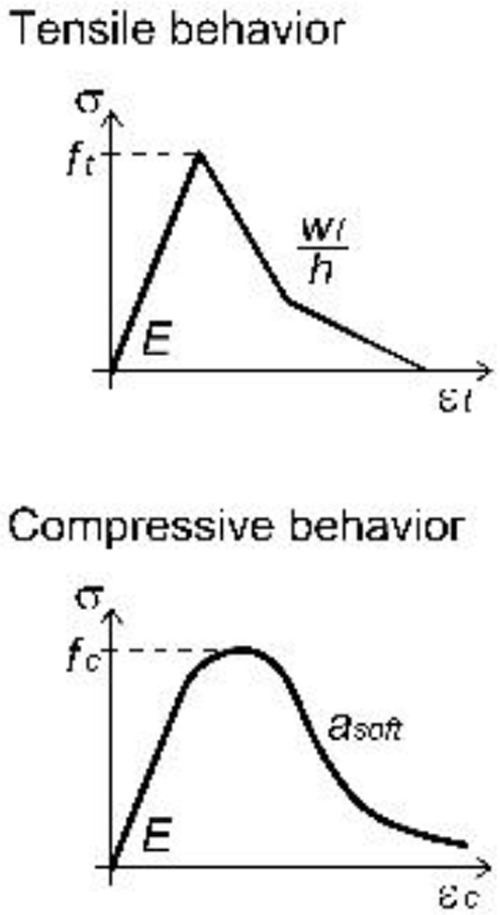
OOFEM material type	*Cdpm2*	
Young modulus *E*	14.4 GPa	
Poisson modulus *n*	0.25	
Self-weight *γ*	20 kN/m ^3^	
Compressive strength *f _c_*	6.29 MPa	
Tensile strength *f _c_*	0.85 MPa	
Dilation ψ	40°	
Softening law	Bilinear	
Hardening parameters *b _h_* , *h _p_*	0. 002, 0.0	
Softening parameters *w _f_/h*, *a _soft_*	0.035, 4	
*f _t1_*, *w _f1_/h*	0.45, 0.0045	
For unspecified parameters, the OOFEM default values are used http:// www.oofem.org/doku.php?id=en:manual		

**Figure 4.**
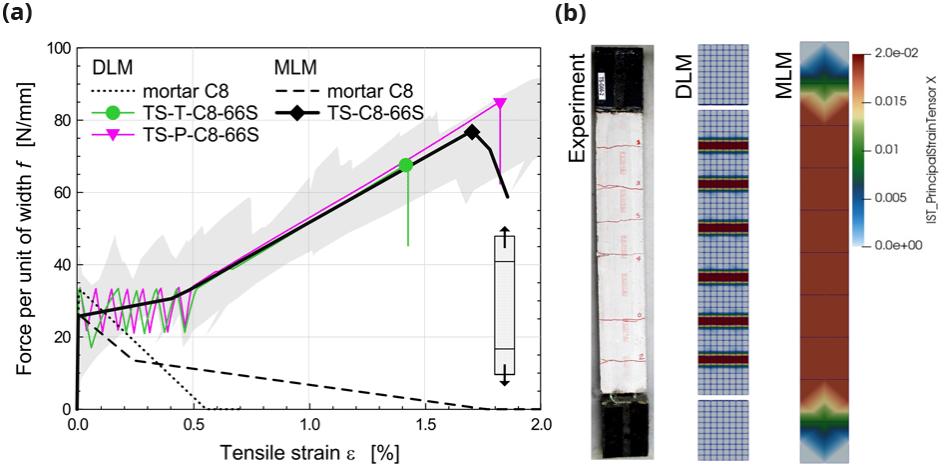
Direct tensile tests on composite reinforced mortar (CRM) coupons: comparison in terms of ( **a**) capacity curves and ( **b**) crack pattern. Acronyms MLM and DLM stand for the multi-layer and the detailed level modelling, respectively.

In Figure 4a, as an example, it is reported the comparison between the outcomes of the DLM (green and pink curves) and the MLM (black curve) when simulating the behavior of CRM coupons subjected to direct tensile tests (id. “TS”). The results are referred to 1220x132x30 mm ^3^ mortar samples (type “C8”) with a 66S GFRP grid embedded and provided with 132 mm long clamping heads. Labels *T* and *P* in DLM curves distinguish the orientation of the GFRP grid (twisted or parallel fiber yarns disposed in the loading direction, respectively). The equivalent tensile strains of the CRM coupon were calculated by dividing the sample elongation for the measurement base length, equal to 860 mm. The principal tensile strains at the peak load are compared in Figure 4b, for the different models. The MLM curve fits well when compared to the DLM results, which were already validated in 22 by comparison with literature experimental outcomes available in 34 (gray shade area). Clearly, the MLM, being smear-cracked, do not allow the detection of the single cracks, nor the respective jagged stress-strain curve characterizing the post-cracking stage, but catches with satisfying approximation the typical trilinear behavior of CRM, as well as the spreading of plasticization over the whole coupon length. Dotted and dashed curves in Figure 4a trace the assumed behavior of the mortar in DLM and MLM, respectively, evidencing the fictitious increase of the fracture energy introduced in MLM to account for the tension stiffening effect. Note also that the mortar tensile strength in MLM was reduced by about 20–25%. in respect to DLM, to fit adequately the mean behavior of the second stage.

**Figure 5.**
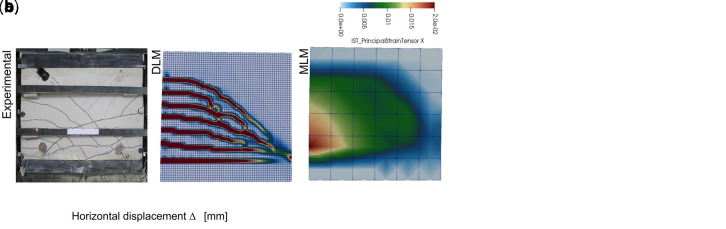
In-plane tests on composite reinforced mortar (CRM) thin slabs: comparisons in terms of ( **a**) capacity curves and ( **b**) crack pattern. Acronyms MLM and DLM stand for the multi-layer and the detailed level modelling, respectively.

A further proof of the reliability of the simplified MLM approach for CRM was achieved by simulating the in-plane shear tests previously carried out on CRM thin slabs (id. “IS”). The tests consisted in 1000x1000x30 mm ^3^ mortar samples (type “C8”) with 66S GFRP grid embedded, provided with top and bottom clamping heads (132 mm high). The outcomes of the DLM (green and pink curves) and the MLM (black curve) simulations, plotted in Figure 5a, are in good agreement (the horizontal displacement was evaluated at the top right corner). Note that the DLM results were already validated in 22 by comparison with experimental outcomes available in 35. Labels *T* and *P* in DLM curves distinguish the orientation of the GFRP grid (twisted or parallel fiber wires disposed in the vertical direction, respectively). The principal tensile strains at the peak load are compared in Figure 5b, for the different models.

Three different testing scales of CRM strengthened masonry were investigated by using the MLM: elementary specimen, structural element and building. As the experimental results available in the literature and used for comparison refer to various masonry types, different parameters necessitated to be set for the simulations: the assumed values are summarized in Table 3. The calibration was based on the outcomes of the available tests on plain masonry samples (tested *e.g.* in compression, diagonal compression, shear compression), trying to reproduce as close as possible the experimental test on plain masonry.

**Table 3.** Main calibrated numerical parameters adopted for the masonry.

MASONRY	B	R	C	S2	B2	B1	Q2	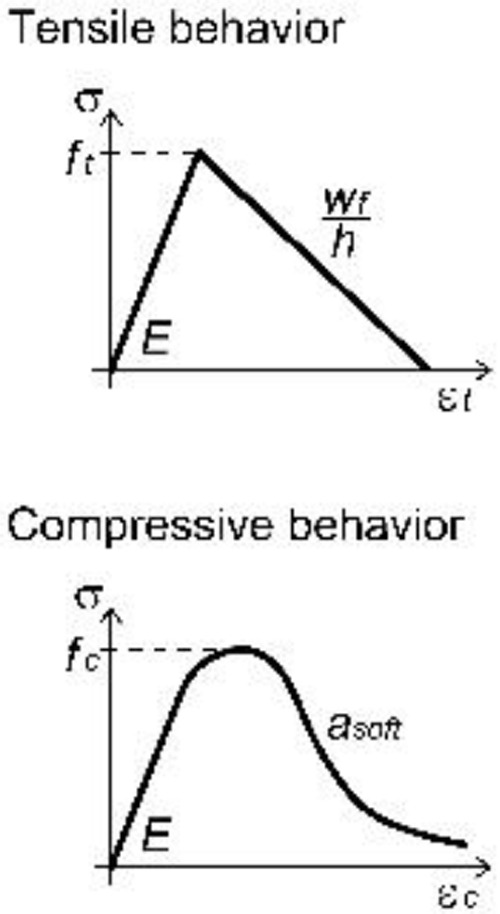
Type	Solid brick	Rubble stone	Cobblestones	Rubble stone	Solid brick	Solid brick	Rubble stone	
Material type	*Cdpm2*	*Cdpm2*	*Cdpm2*	*Cdpm2*	*Cdpm2*	*Cdpm2*	*Cdpm2*	
Young mod. *E*	4.27 GPa	2.43 GPa	1.26 GPa	4.50 GPa	3.97 GPa	4.91 GPa	3.71 GPa	
Poisson modulus *n*	0.45	0.45	0.45	0.45	0.45	0.45	0.45	
Self-weight *γ*	18 kN/m ^3^	21.0 kN/m ^3^	19.0 kN/m ^3^	21.0 kN/m ^3^	18 kN/m ^3^	18 kN/m ^3^	21.0 kN/m ^3^	
Compressive strength *f _c_*	5.12 MPa	2.13 MPa	1.04 MPa	2.60 MPa	2.95 MPa	3.83	2.0 MPa	
Tensile strength *f _c_*	0.320 MPa	0.208 MPa	0.089 MPa	0.084 MPa	0.100	0.162	0.069 MPa	
Dilation ψ	30°	35°	40°	40°	40°	40°	40°	
Softening law	Linear	Linear	Linear	Linear	Linear	Linear	Linear	
Hardening parameters *b _h_* *h _p_* *k _init_*	0.003 0 0.3	0.006 0 0.3	0.006 0 0.3	0.022 0 0.15	0.015 0 0.15	0.015 0 0.15	0.022 0 0.15	
Softening parameters *w _f_/h* *a _soft_*	0.0001 5.0	0.004 15.0	0.004 10.0	0.004 5.0	0.003 8.0	0.004 8.0	0.004 5.0	
For unspecified parameters, the OOFEM default values are used http://www.oofem.org/doku.php?id=en:manual								

## Elementary specimen level

**Figure 6.**
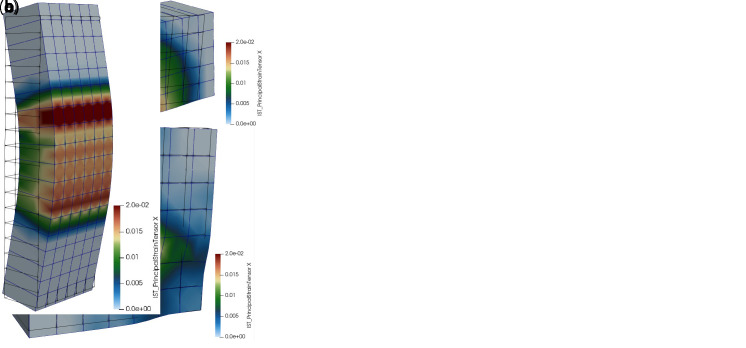
The multi-layer modelling (MLM) simulations of elementary specimens: ( **a**) diagonal compression, ( **b**) in-plane and ( **c**) out-of-plane bending tests (the principal tensile strains on the displayed surface are visualized).

The application of the MLM approach to the simulation of elementary masonry specimens strengthened with CRM was at first performed, so to check its capability in detecting the main failure mechanisms of historic masonry under lateral loads, namely diagonal-cracking, in-plane bending and out-of-plane bending. The simulations concerned, respectively, diagonal-compression tests, three-point in-plane bending tests and four-point out-of-plane bending tests ( Figure 6). For each model, comparison was made with experimental tests available in the literature and carried out under loading-unloading procedures (the backbone capacity curves were considered).

**Figure 7.**
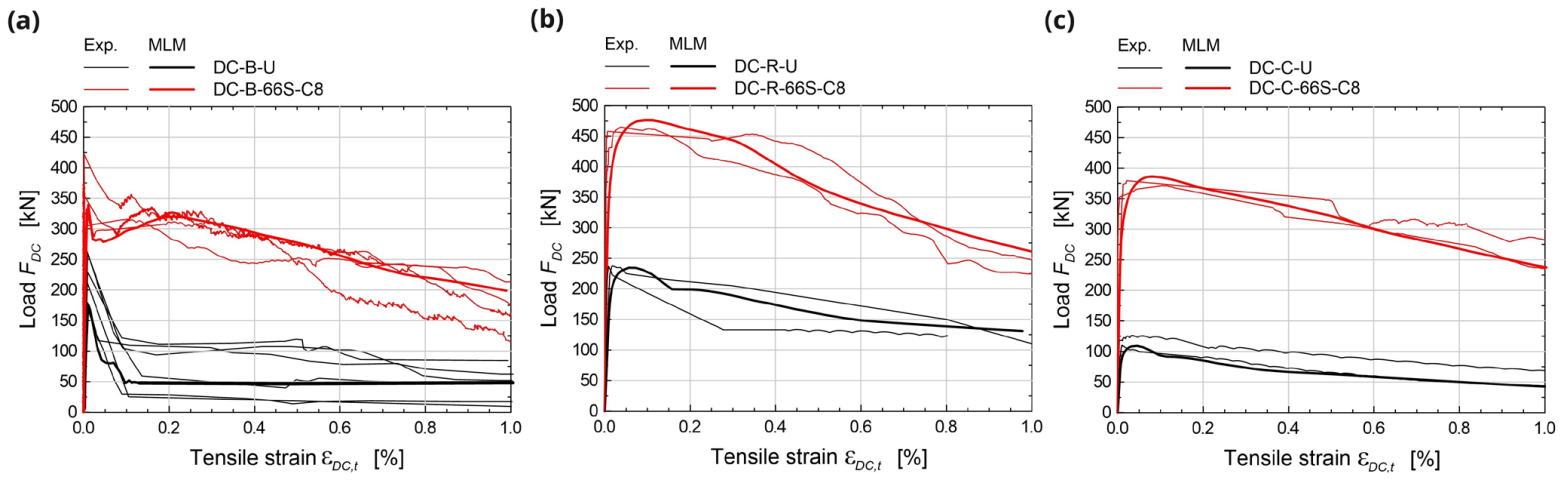
Diagonal compression tests: comparison between the multi-layer modelling (MLM) and experiments (Exp.); *ε _DC,t_* is the tensile strain along the sample diagonal orthogonal to the loading direction (evaluated on a base length of 1100 mm).

For the simulations of diagonal-compression tests ( Figure 6a), square masonry panels (1160 mm side) were modelled. More details about the experimental setup and outcomes used for comparison can be taken from 36. According to the experimental setup, the nodes at one corner (bottom-right) were pinned and the diagonal displacement was applied at the nodes at the opposite one (top-left). Moreover, the nodes close to the corners were forced with the same boundary conditions, so to account for the presence of the stiff steel brackets actually introduced to apply the load. The comparisons between experimental and numerical results are reported in Figure 7 in terms of capacity curves expressing the diagonal load *F _DC_* varying the tensile strain *ε _DC,t_* along the sample diagonal orthogonal to the loading direction (base length about 1100 mm). The results concerned different masonry types: solid brick B (250 mm thick), rubble stone R and cobblestone C (400 mm thick). The masonry characteristics, reported in Table 3, were the same already calibrated when developing the DLM approach ^28^. In summary, the masonry tensile strength *f _t_* was calculated from the peak load obtained from experimental diagonal compression tests on unstrengthened samples; the softening parameters fit with the mean experimental post-peak behavior. The masonry Young’s modulus and compression parameters fit with the mean results of experimental compression tests on plain masonry wallets. The MLM provided capacity curves generally comparable to the experimental ones. The failure originated diagonally, in the central portion of the sample and propagated with some deviation from the loaded corners, as the presence of the steel devices confined somehow the masonry, creating pushing wedges ( Figure 6a – the principal tensile strains of the surface are displayed).

**Figure 8.**
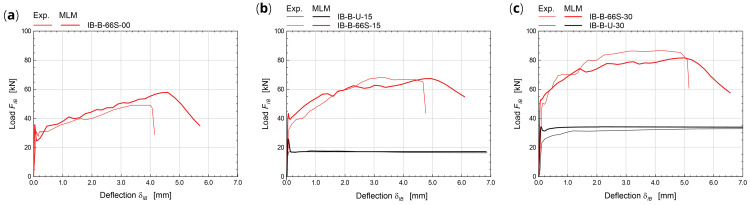
In-plane bending tests: comparison between the multi-layer modelling (MLM) and experiments (Exp.).

The numerical simulations concerning elementary samples subjected to in-plane bending tests ( Figure 6b) referred to rectangular masonry wallets made of solid bricks (type “B”), 780 mm wide, 380 mm tall and 250 mm thick. More details about the experimental tests taken for comparison can be found part in 37 and part in 28. Three-point bending was created by lying down the samples on a span of 680 mm and assigning the vertical translation constrains at the support nodes, at the intrados. Then, opposite horizontal forces were applied at the lateral ends nodes, to introduce a constant axial stress. Lastly the vertical displacement of the nodes at the extrados, at the mid-span, were introduced and gradually incremented. The experimental-numerical comparisons are reported in Figure 8, for different axial stress levels (0, 0.15 and 0.30 MPa), in terms of capacity curves expressing the vertical load, *F _IB_*, varying the mid-span deflection at the intrados, *δ _IB_*. Considering the huge coarseness of the mesh for such a reduced sample size, the model satisfactory reproduces the global behavior of CRM strengthened samples, with failure originating at the intrados, at mid-span, and gradually spreading out, mostly in the lower part ( Figure 6b).

**Figure 9.**
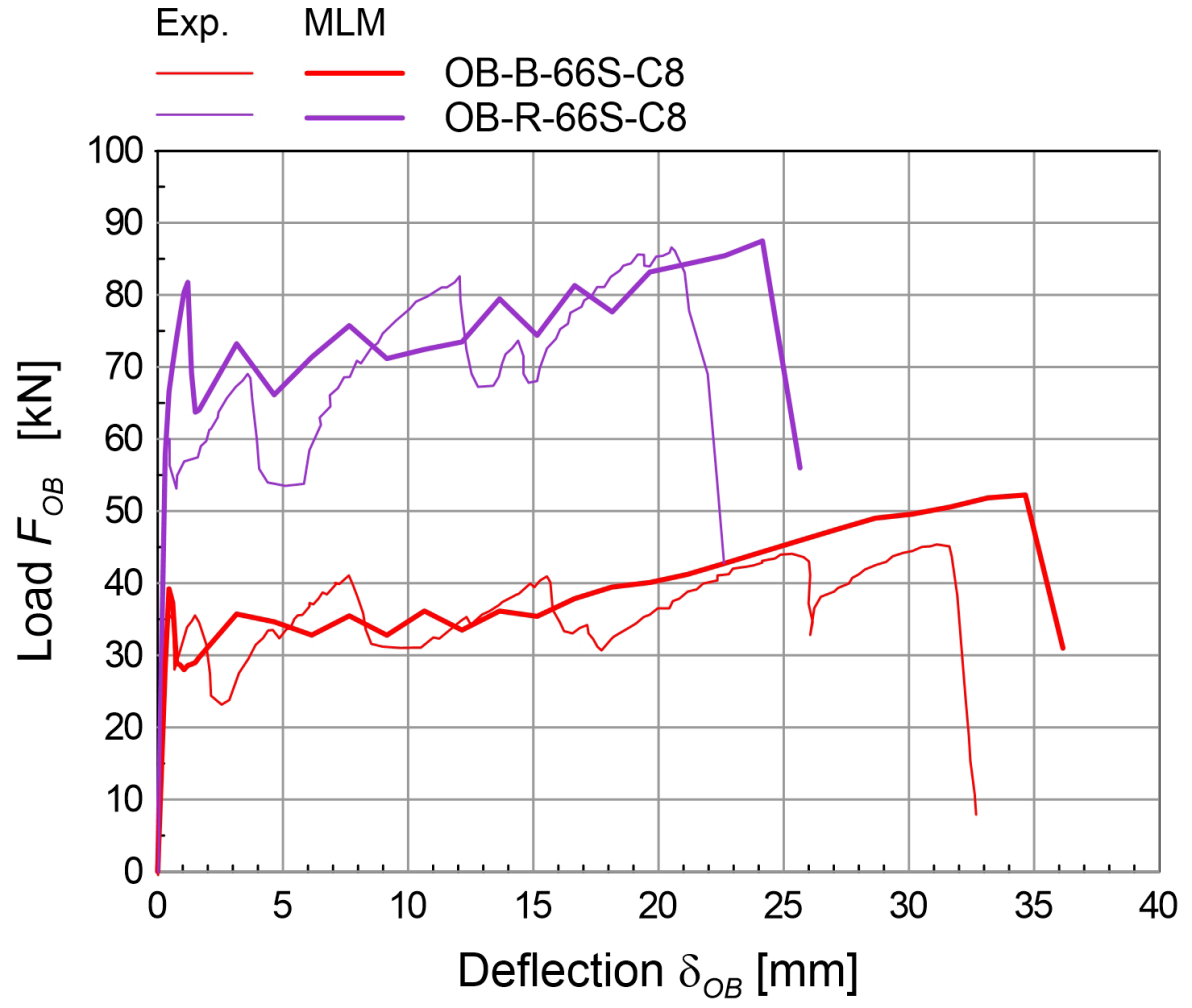
Out-of-plane bending tests: comparison between the multi-layer modelling (MLM) and experiments (Exp.).

The simulations of the four-point bending tests ( Figure 6c) concerned masonry samples 3000 mm tall and 1000 mm wide. The samples were placed on a steel support providing the vertical linear constraint, at the mid-thickness; horizontal steel beams provided the horizontal linear constraints at the top and at the bottom of the sample front side; two equal, horizontal forces were applied at the thirds of the height, at the back side. More details about the experimental tests taken for comparison can be found in 38. To reproduce the boundary conditions of the experimental test, vertical translations constraints were applied at the base nodes, at the mid-thickness nodes, and horizontal translations constraints at the top and at the bottom of the front side. The specimen self-weight was introduced in the vertical direction and two equal, horizontal forces were applied at thirds of the total height, at the back side, and then scaled so as to increase progressively the wall deflection. Solid brick masonry B (250 mm thick) and rubble stone masonry R (400 mm thick) were considered ( Table 3). The experimental-numerical comparisons are reported in Figure 9, in terms of capacity curves expressing the horizontal load *F _OB_* varying the mid-span deflection at the front side *δ _OB_*. The failure of the CRM strengthened samples originated at the front-side, at two-thirds of the height, and gradually spread out on a wider central area; the collapse of strengthened samples was due to the failure of the GFRP reinforcement ( Figure 6c). Since the goal of the MLM approach is application at a large scale, the detected discrepancies in the capacity curves are considered acceptable at the small scale of elementary samples.

## Structural element level

A series of experimental cyclic tests, available in the literature, was recently performed to assess the effectiveness of the CRM strengthening technique when applied on both sides or one side of masonry structural elements. In particular, the laboratory experimentations concerned full scale piers and spandrel samples, representing the wall portions between adjacent openings arranged, respectively, horizontally (at the same storey level) or vertically (different, contiguous storeys). The MLM was thus applied to the simulation of such tests, so to assess, through comparison with the experimental outcomes, its validity at the structural element level. The description of the main features of the numerical models and results and the comparison with the previous experimental evidences are described and commented in the following sections.

### Masonry piers

**Figure 10.**
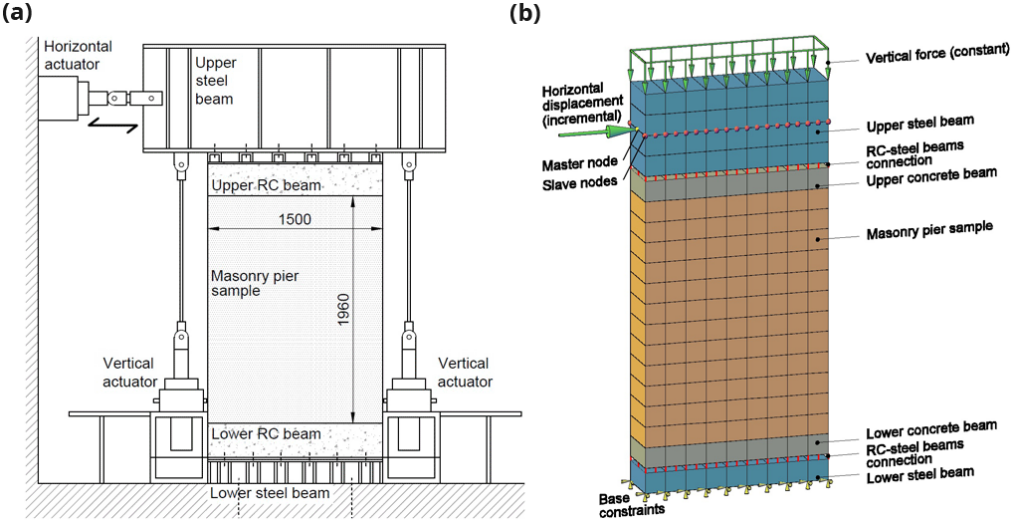
In-plane tests on piers: schematization of ( **a**) the experimental setup and ( **b**) the multi-layer modelling (MLM). Acronym RC stands for reinforced concrete.

**Figure 11.**
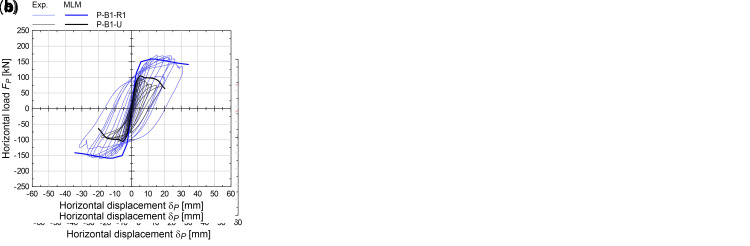
In-plane tests on piers: comparison between the multi-layer modelling (MLM) and experimental capacity curves (Exp.).

The masonry pier samples (id. “P”) consisted in rectangular wall portions having a width of 1500 and a height of 1960 mm ( Figure 10a). The test setup is schematized in Figure 10a: each sample was built in the testing laboratory on a reinforced concrete (RC) beam 1500 mm wide, 300 mm high and with a thickness equal to that of the plain masonry. The RC beam was bolted to a stiff steel beam fixed to the laboratory floor. On the top of the sample, another RC beam, of the same dimensions, was positioned and then bolted to the upper stiff steel beam of the testing apparatus. At the lateral extremities of the upper steel beam, two electro-mechanical actuators, connected to the floor, were installed to control the amount of vertical axial load and the rotation at the top. During testing, they were governed so as the applied axial load was maintained constant during the tests (axial stress level equal to 0.5 MPa) and the rotations of the upper steel beam were avoided. A third actuator, positioned at one side of the upper steel beam (left side), at its mid-height, applied the lateral loading cycles at increasing displacements. Three masonry types were built: double wythe rubble stone masonry, 350 mm thick and solid brick masonry 250 mm thick, built up in single wythe and in double wythe. They corresponded, in terms of type and mean mechanical properties (strengths and stiffness), to the types S2, B1 and B2 of Table 3, respectively. For each masonry type, one sample was tested unstrengthened (U), as reference, one with the CRM applied at both sides (R2) and one with CRM at one side only (R1), according to features the described in the section “Strengthening with CRM”. In exception, B1 masonry did not have the R2 configuration. The experimental results are reported with thin lines in the graphs of Figure 11, in terms of applied horizontal load *F _P_* varying the horizontal displacement of the control point *δ _P_*, at the upper-right corner of the masonry sample. Further details and discussion about the experimentations can be found in 39, 40.

The numerical model is schematized in Figure 10b: the 20 nodes brick elements in blue color represent the steel beams, those in gray the RC beams and those in orange the masonry sample. The first row of brick elements was fixed at the base, while the steel and the RC beams were connected by means of vertical elastic links; they were introduced to simulate the slight but not negligible vertical displacements actually monitored in those areas through dedicated potentiometer transducers. Differently, a perfect bond was assumed between the masonry pier and the RC beams. When considering CRM strengthened masonry samples, the multi-layer approach ( Figure 3) was assumed also for the RC beams, since they were also involved by the CRM application, to simulate the actual continuity of the strengthening intervention at the pier ends in a building. To avoid the upper steel beam rotations, a master node was selected in correspondance of the horizontal actuator and all the other nodes at that height were forced to have the same translations. To simply reproduce connectors and diatones, axial rigid links connecting the nodes at the opposite wall faces were introduced. The self-weights of the sample and of the experimental apparatus were considered; moreover, the additional vertical load applied by the vertical actuators (corresponding to an axial stress level equal to 0.5 MPa) was distributed at the top of the upper steel beam and maintained constant. Then, a horizontal load was applied at the height of the actuator and was varied so as to increase monotonically the horizontal displacement of the control point.

The parameters of the plain masonry, in terms of strength and stiffness ( Table 3) were set starting from of the values suggested in the commentary of the Italian building code ^41^ for “stone masonry with good texture” (for S2) and “solid brick” masonry types (for B2 and B1). In particular, for S2, the minimum values of the suggested ranges were set. Differently, for the solid brick masonry types, the compressive strength and the mean elastic modulus were calculated by performing direct linear interpolation within the range, starting from the calculated tensile strength, *f _t_*. This latter was calculated from the experimental shear compression tests results on plain masonry, by applying the well-known Turnšek and Čačovič formulation ^42^: *F _P,h_* = *l·t·f _t_* / *b·*(1+ *σ _0_*/ *f _t_* ) ^1/2^. *F _P,h_* is the pier lateral resistance, *l*· *t* its cross section, *b* the slenderness ratio (1≤ *b*= *h/l*≤1.5), *h* the height and *σ _0_* the axial stress level (0.5 MPa). For *F _P,h_*, the mean resistance obtained from the bi-linearization of the backbone experimental curves in the two directions was considered. For all masonry types, the softening parameters, indicated in Table 3, set so to fit the experimental results of the shear-compression tests ^39, 40^ recently performed on plain masonry (black capacity curves plotted in Figure 11). In the simulations, the stress-strain uniaxial compressive law was linear elastic till achieving 15% the strength ( *k _init_* = 0.15 in Table 3); then it prosecuted with a parabolic trend, so to account for a progressive stiffness degradation. Thus, the used value of Young modulus was set equal to 3 times the value provided in 41, which refers to an average modulus, accordingly to a simplified, linear elastic behavior up to the peak. Note that, due to the hypothesis of homogeneous isotropic material, the masonry behavior is assumed constant in all directions and, thus, regardless the orientation in respect to the bed joints. Although this simplified hypothesis would not be exactly adequate to accurately simulate the behavior of the unreinforced masonry, it is considered an acceptable approximation for the purposes of the MLM model, focused on reinforced masonry, whose behavior is mainly dominated by the CRM tensile capabilities.

**Figure 12.**
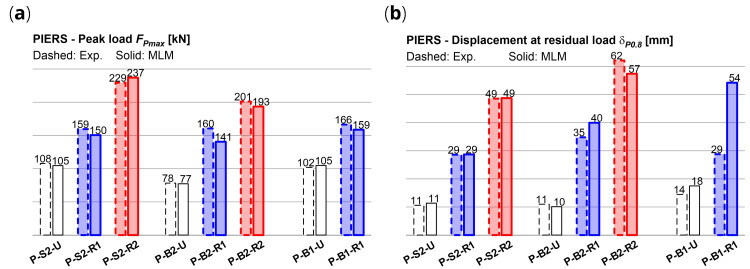
In-plane tests on piers: numerical and experimental ( **a**) peak load and ( **b**) residual displacement. Acronyms MLM and Exp. stand for the multi-layer modelling and the experimental results, respectively.

The numerical results are reported with thick lines in the *F _P_*- *δ _P_* graphs of Figure 11, in comparison with the experiments. It generally emerged a good accordance of the numerical capacity curves of the CRM strengthened sample with the envelope of the cyclic experimental ones in terms of global trend. The errors in terms of predicted peak load *F _Pmax_* in the strengthened sample ranged between -12.4% and +3.3% ( Figure 12a); the displacement *δ _0.8_*, associated to a conventional residual load (set equal to 0.8 *F _Pmax_*) were predicted with errors generally ranging from -7.6% and +14.8%. ( Figure 12b). Exception for P-B1-R1, in which *δ _P0.8_* was overestimated by +88%. However, in this case, it has to be considered that the drop down of the experimental curve after 25 mm was related to the unexpected local crushing occurred at the ends of the upper RC beam.

**Figure 13.**
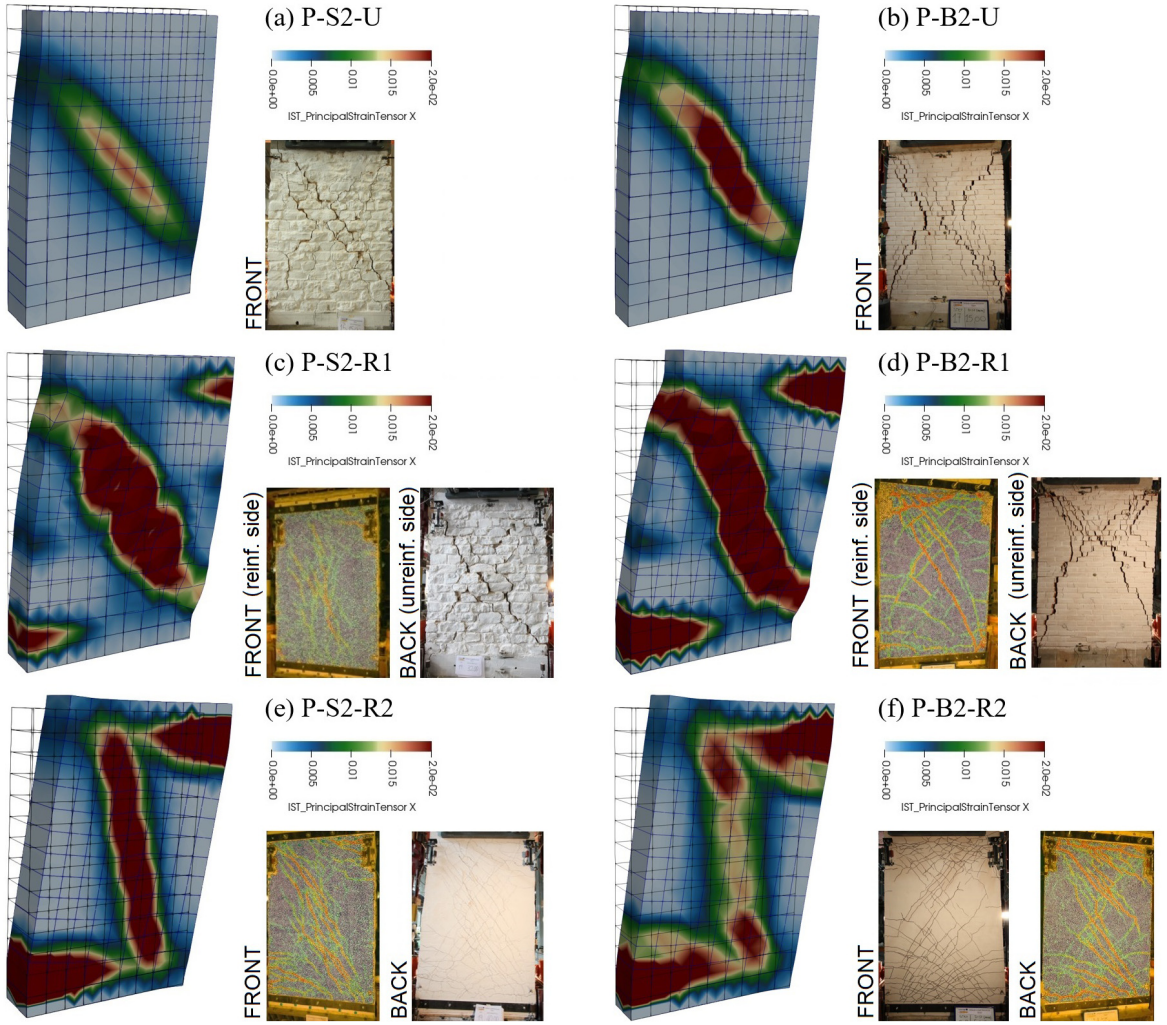
In-plane tests on piers: comparison between numerical and experimental damage pattern (the principal tensile strains on the displayed surface are visualized).

Also the damage patterns resulted in agreement with the failure modes detected in the experiments ( Figure 13). In fact, whilst the unstrengthened samples clearly failed for diagonal cracking (high tensile strains located along the diagonal - Figure 13a–b), mixed damaging occurred in the strengthened ones, in agreement with the experimental evidences. In particular, the damaging associated to the in-plane bending mechanism (high tensile strains localized at the extremities) also appeared. For samples strengthened at one side only (R1) diagonal cracking still remains the dominant failure mode ( Figure 13c–d), while combined diagonal cracking and bending failure modes ( Figure 13e–f) affected samples strengthened at both sides (R2).

### Masonry spandrels

**Figure 14.**
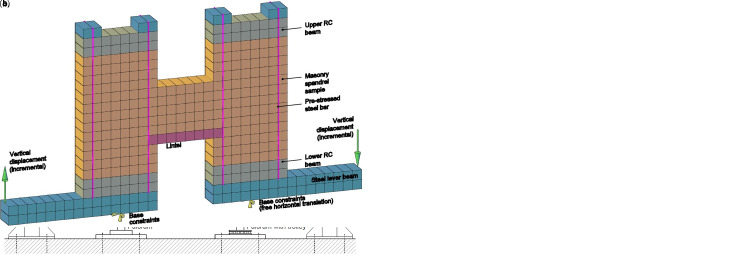
In-plane tests on spandrels: schematization of ( **a**) experimental setup and ( **b**) the multi-layer modelling (MLM).

The experimental samples to test the behavior of masonry spandrels (id. “S”) had an H-shape with global width and height of 3890 mm and 2190 mm, respectively. The spandrel was 1050 mm wide and 1170 mm tall, while the two lateral walls were 1420 mm wide and 2190 mm tall and had at the base and top RC beams (1420 mm long and 350 mm high). Each lateral wall was located on a stiff steel lever beam, with fulcrum at the mid-width of the wall ( Figure 14a). The horizontal sliding was also allowed for the right beam. Each wall was loaded vertically under axial constant stress level (~0.33 MPa) by means of two couples of tightened steel bars connecting the top RC beam with the bottom lever beam. A vertical hydraulic actuator was installed over the external end of each lever beam. During the test, the couple of vertical actuators imposed vertical displacements of opposite amplitude, so that lateral walls were cyclically rotated during the tests and shear forces were induced on the connecting spandrel. Three cycles were performed for each step amplitude. Further details and discussion about the experimentations can be found in 39 and in 43.

**Figure 15.**
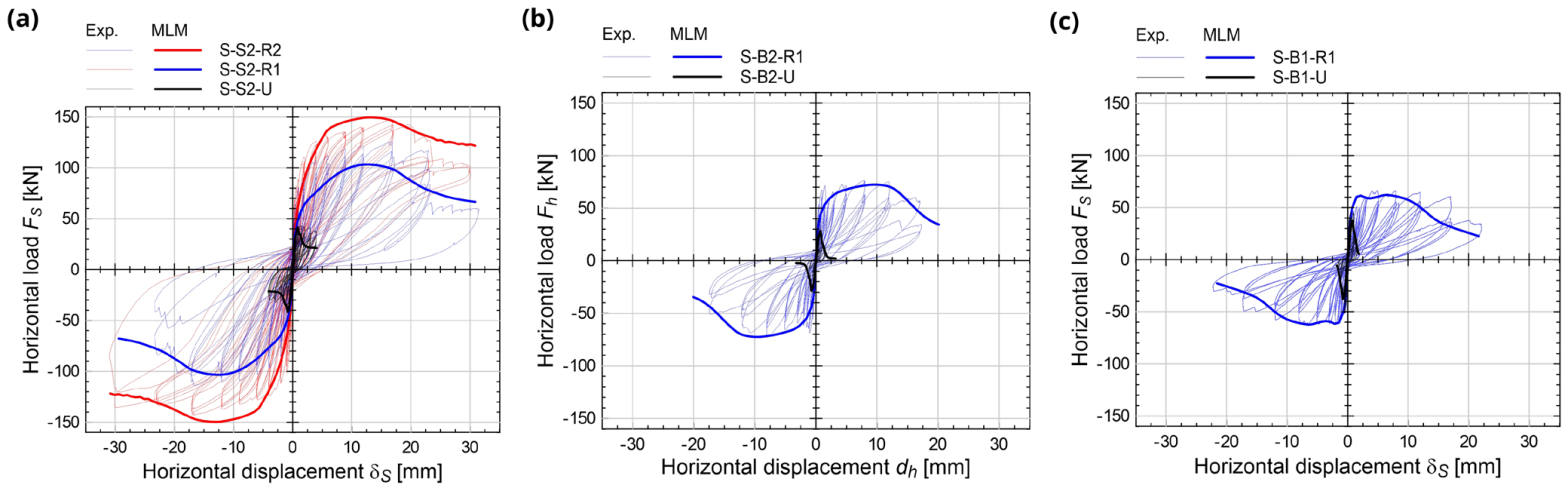
In-plane tests on spandrels: comparison between the multi-layer modelling (MLM) and the experimental capacity curves (Exp.).

Like the pier ones, the experimental samples were built in three masonry types: double wythe rubble stone masonry, 350 mm thick, and solid brick masonry 250 mm thick, built up in single wythe and in double wythe. They corresponded, in terms of type and mean mechanical properties (strengths and stiffness), to the types S2, B1 and B2 of Table 3, respectively. The spandrels made of rubble stone were provided by timber lintels, while flat masonry arches were created for the samples made of solid bricks. The samples were tested unstrengthened (U), then the cracks were repaired through grout injection and the masonry was retrofitted with CRM at one side (R1) or, just for the stone masonry, at both sides (R2), in accordance to the features described in the section “Strengthening with CRM”. The experimental results are reported with thin lines in the graphs of Figure 15, in terms of average reaction load at the fulcrums, net of the self-weight, *F _S_*, at the varying the displacement *δ _S_*, evaluated as the gap between the vertical displacements monitored in the inner corners of the lever beams.

The numerical model is schematized in Figure 14b: the 20 nodes brick elements in blue color represent the steel beams, those in gray the RC beams, those in orange and red the masonry sample and the lintel. When modelling the strengthened samples, the multi-layer approach ( Figure 3) was assumed also for the lintels, since they were also involved by the CRM application, as actually occurs in buildings. Truss elements represented the steel bars. To simply reproduce connectors and diatones, axial rigid links linking the nodes at the opposite wall faces were introduced. The nodes in correspondance of the left lever fulcrum were pinned, while in those at the right one the horizontal translation was left free. The self-weight of the sample was at first applied; then, the pre-compression applied by the tightened bar was considered by means of an equivalent temperature variation. The vertical displacement at the external ends of the two lever beams was incremented monotonically, so that the displacement at the right had equal magnitude and opposed direction to that at the left.

**Figure 16.**
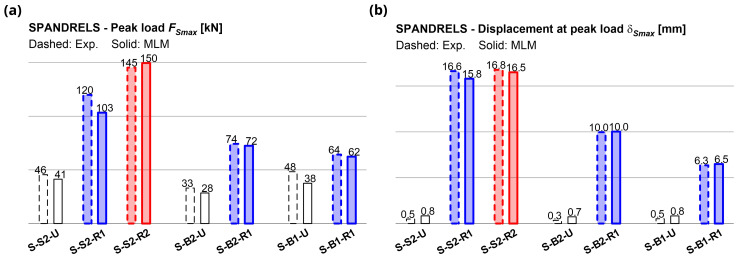
In-plane tests on spandrels: numerical and experimental ( **a**) peak load and ( **b**) displacement at peak. Acronyms MLM and Exp. stand for the multi-layer modelling and the experimental results, respectively.

**Figure 17.**
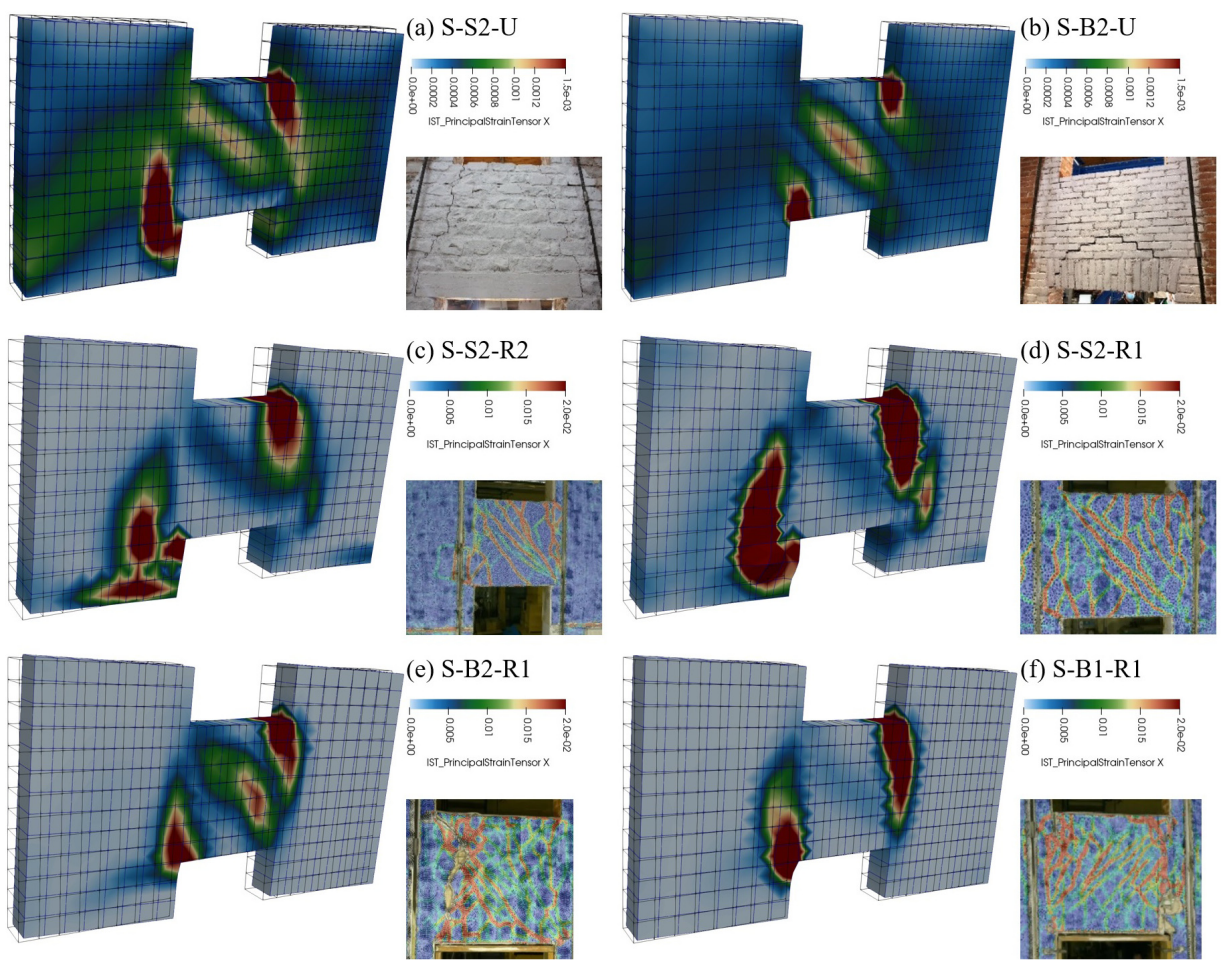
In-plane tests on spandrels: comparison between numerical and experimental damage pattern (the principal tensile strains on the displayed surface are visualized).

The numerical *F _S_*- *δ _S_* capacity curves are reported with thick lines in Figure 15, in comparison with the experimental ones. The errors in terms of predicted peak load *F _Smax_* in strengthened samples ranged between -13.8% and +2.9% ( Figure 16a); those in terms of displacement at the reaching of *F _Smax_*, *δ _Smax_*, between -5.0% and +2.2% ( Figure 16b). The coherence of the predicted damage patterns with the experimental evidences was also confirmed. In the unstrengthened sample S2 ( Figure 17a), the higher tensile strains localized vertically, at the spandrel extremities (flexure-dominated failure), while in B2 masonry ( Figure 17b), higher tensile strains were attained also along the spandrel diagonal (combined shear-bending failure); sample B1 exhibited an in-between behavior, but the failure was governed by the bending mechanism. In the strengthened samples, high levels of tensile strain were reached both at the spandrels extremities and along diagonals. However, for S2 and B1 masonry, a flexure-dominated collapse emerged ( Figure 17c,d,f) while, for B2 masonry, the failure was due to combined shear-bending mechanism ( Figure 17e).

## Building level

**Figure 18.**
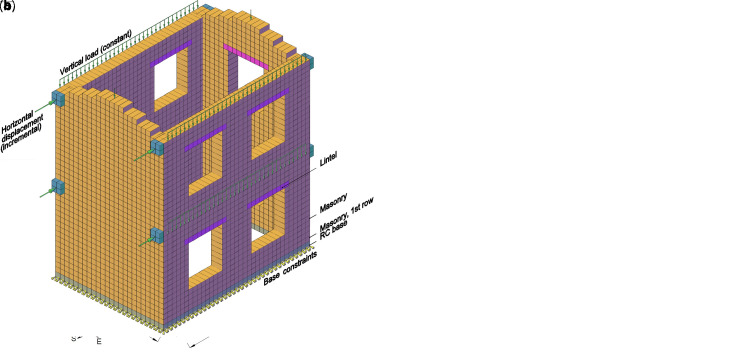
Pushover test on building: schematization of ( **a**) the experimental setup and ( **b**) the multi-layer modelling (MLM). Acronym RC stands for reinforced concrete.

To assess the reliability of the MLM at the building level, the experimental results of recently performed pushover tests on a two-story masonry house strengthened through CRM were considered ^39, 44^ (identification label “GB” for the Global Building model). The main characteristics are reported in Figure 18a: the structure was made of double wythe, rubble stone masonry walls (350 mm thick) and had plan dimensions of 5750x4350 mm ^2^ and a height of 6000 mm. The building had a wooden floor with unidirectional joists (120x160 mm ^2^, 600 mm spaced) arranged along the East-West direction and provided by nailed timber boards (25 mm thick); a distributed mass was added on the boards, to simulate the carried masses. The double pitch, timber roof was made of joists (100x140 mm ^2^, 570 mm spaced) laid on the central ridge beam (200x320 mm ^2^) and on the longitudinal walls and was covered by nailed timber boards (25 mm thick) and clay tiles. The overall gravity load was 71.5 kN at the 1 ^st^ floor and 51.7 kN at the roof level. The horizontal cyclic load was applied along the North-South direction, governing the amplitude of the mean horizontal displacement of the control points (top corners of the North wall). Two cycles were performed for each step amplitude. The load was applied by means of two mechanical actuators, located in the vicinity of the South wall. Pinned nodes steel frames, connecting the actuators with the building, allowed the load distribution between the loading point on the first floor and the one at the top, accordingly to a prescribed distribution (proportional to the first vibration mode). A system of steel ties, let pushing on the South wall side, when loading from South to North, while on the North wall side, when loading from North to South.

**Figure 19.**
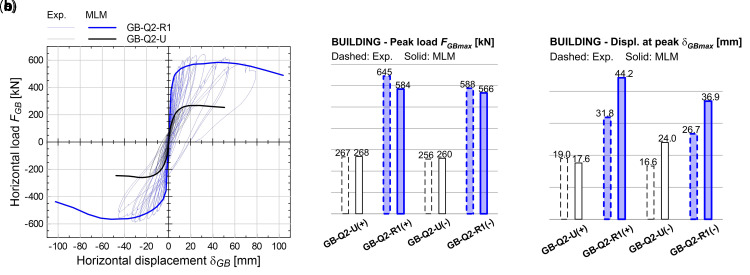
Tests on building: comparisons of ( **a**) capacity curves, ( **b**) peak load, ( **c**) displacement at peak. Acronyms MLM and Exp. stand for the multi-layer modelling and the experimental results, respectively.

The building was at first tested unstrengthened, then retrofitted with CRM at the external side only (according to the features described in the section “Strengthening with CRM”) and tested again. GFRP angular grid elements (330 mm side, 66x66 mm ^2^ grid pitch) were used to ensure the reinforcement continuity at the four corners, along the whole building height. To provide the connection with the RC fixed foundation, vertical steel threaded bars (φ8, *f _yd_* = 200 MPa, 3/m) were embedded in the mortar coating (for a length of 400 mm) along the building perimeter and fixed through injection into holes drilled in the RC foundation (250 mm depth). The experimental results are reported with thin lines in the graphs of Figure 19a, in terms of global horizontal force, *F _GB_*, varying the mean horizontal displacement of the two control points, *δ _GB_*. Further details and discussion about the experimentations can be found in 39, 44.

The numerical model ( Figure 18b) was created according to the features already described for the pier and spandrel samples (section “Structural element level”). Moreover, the RC fixed foundation was reproduced with a row of 20-nodes brick elements, with rigid behavior, pinned at the base, located at the bottom of the building. To account for the plaster interruption and for the presence of the steel bars at the base, a different multi-layered cross section was defined for the brick elements of the second row of the model. In particular, a reduced tensile strength (0.15 MPa, instead of 0.85 MPa) was assigned to the layer representing the plaster ( Figure 3b). Furthermore, the characteristics of the unitary thickness layer were modified taking into account the steel bars, instead that of the GFRP grid (layer equivalent properties: simplified linear elastic-plastic behavior, with Young’s modulus *E* = 20.8 GPa, yielding strain *ε _y_* = 0.48% and ultimate strain *ε _u_* = 26.8%). The presence of the GFRP angular grid elements at the corners was accounted by considering a doubled thickness for the layer representing the reinforcement. The masonry stiffness and strength characteristics (Q2 in Table 3) were set by scaling of about 80% that of type S2. This to consider the actual reduction emerged in the experimental compressive strength of the building masonry, in respect to that of the piers and spandrel samples. The masonry self-weight was applied. Due to the high deformability of the floor and roof, they were simply modelled just in terms of additional vertical load applied on the masonry walls. Then, the displacement at the four loading points was incremented monotonically, according to the experimental distribution. Two different simulations were carried out for the positive and negative loading direction.

The numerical results are reported with thick lines in Figure 19a, in terms of *F _GB_*- *δ _GB_* capacity curves: even based on non-linear static analysis, a good agreement with the cyclic loading experimental envelope curves emerged. The error in terms of peak load, *F _GBmax_* ( Figure 19b), in the strengthened configuration, is about -9.5% in the positive loading direction and -3.7% in the negative one, while the displacements at peak load, *δ _GBmax_* ( Figure 19c), show some overestimation (about +38%). But this is related to the smoother ridge of the numerical curve, in respect to the experimental one. Looking at the positive loading direction, the two curves tend to get closer.

**Figure 20.**
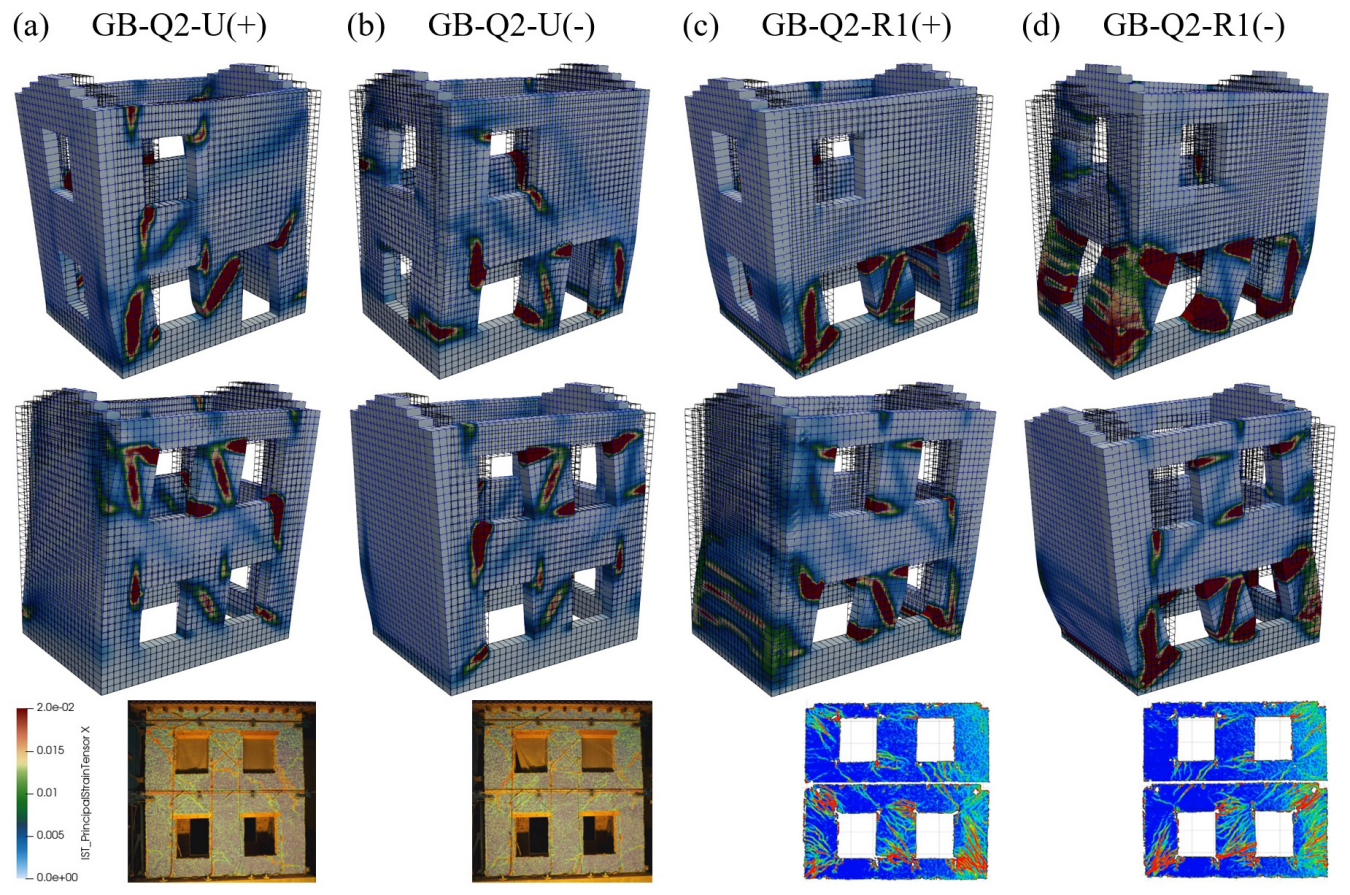
Pushover tests on building: comparison between numerical and experimental damage patterns at the ultimate displacement reached in the simulations or experiments (the principal tensile strains on the outer surface are visualized).

The damage patterns are reported in Figure 20. The activation of the different collapse mechanisms can be clearly distinguished and are globally in agreement with the experimental damage modes. In the unstrengthened configuration, both in the shear walls (East end West) exhibited the activation of the diagonal failure in some piers at the ground floor. Also in the masonry panel above the door some shear cracking occurred. Moreover, a mixed shear-flexure collapse activated in the piers of the first floor of the East wall and the spandrels of the first floor. In the strengthened configurations, the damage mainly focused at the ground floor, with mixed shear-flexure mechanism. Moreover, horizontal cracks appeared also in the gable walls, which, due to the tensile resistance of the GFRP grid and the connection with the foundation, contributed to provide some additional resistance against the lateral action.

## Conclusions

The article deals with the evaluation of the global effects of TRM interventions on historic masonry structures, focusing on the identification a reliable but computationally efficient numerical strategy. In particular, a multi-layer numerical modelling approach, MLM, was developed. It was based on 20-nodes brick elements composed by a sequence of through-the-thickness plies representing the masonry, the mortar coating and the fiber-based reinforcement. Nonlinear-static analyses were carried out considering the material nonlinearities (cracking and crushing, for the masonry and the mortar coating, and tensile failure of the reinforcement). The material characteristics were deduced from previous experimental evidences on individual components and on CRM coupons subjected to direct tensile tests. The simplified assumption of perfect bond among layers was considered.

The results of some tests on CRM strengthened masonry available in the literature were considered to assess the model reliability at three different scale levels, of increasing complexity: elementary specimen, structural element and building. The results were compared in terms of capacity curves (resistance vs. displacement) and collapse mode. The former level was aimed at the individual recognition of the different, typical failure modes of masonry and the MLM was confirmed capable to notice the diagonal cracking mechanism (by means of diagonal compression tests), in-plane bending mechanism (through in-plane, three point bending tests) and out-of-plane bending mechanism (by out-of-plane, four point bending tests). The intermediate level focused on the performances of actual structural elements in buildings, such as piers and spandrels, in which the MLM was proved able to detect the activation also of mixed failure modes that occurred in many cases. The latter level, at the building scale, showed the reliability of the MLM also in the global analysis, where different resistant elements are combined and interact each other to provide the structure response. The discrepancies in comparison with experimental tests were found to be acceptable at the different scale levels and were attributed to uncertainties in the materials properties (scatter in respect to the nominal values assumed numerically), possible cumulative damage (neglected in the monotonic simulations), a not precise "displacement control" in the experimental tests, the reduced numbers of experimental test available for each configuration.

Given the coarse mesh size and the smear plasticization assumption, the MLM is clearly not suitable for the rigorous reproduction of individual cracks, for which more accurate but computationally heavier models, such as the DLM, should be used. However, it represents a good compromise between the goal to grasp the structural performances at the wide scale, including failure modes, and the analysis optimization. Clearly, the simplified assumption of perfect bond among layers has to be ensured. For example, by respecting the minimum bond lengths, the limits on reinforcement ratio and the number of transversal connections. The calibration of such requirements can be achieved by experimental characterization tests ( *i.e.* direct tensile tests and shear-bond tests) and/or through simulations with the DLM on small samples. Alternatively, it is possible to intervene on the MLM by limiting the ultimate deformation of the reinforcement (when the debonding anticipates the reinforcement failure) and/or the compressive strength of the mortar (so to account for its buckling in the most compressed areas).

Ongoing research concerns an extensive sensitivity analysis with MLM at the structural element level, to provide a robust database for the behavior estimation of piers and spandrels. It will thus be possible to define a simplified bilinear behavior for these elements, so to be used for fast global pushover analysis of CRM strengthened masonry structures, by means the equivalent frame method based on lumped plasticity. The reliability of such models could be assessed by comparison with MLM applied at the building scale.

## Ethics and consent

Ethical approval and consent were not required

## Data Availability

Experimental data for each section was taken from existing literature. Readers and reviewers are able to access this data upon request to the author, if they do not have direct access: - Experimental results of direct tensile tests on CRM coupons ^34^ - Experimental in-plane shear tests on CRM thin slabs ^35^ - Experimental diagonal compression tests on elementary masonry samples ^36^ - Experimental in-plane bending tests on elementary masonry samples, part in 37 and part in 28 - Experimental out-of-plane bending tests on elementary masonry samples ^38^ - Experimental tests on structural elements (piers, spandrels) and on pilot building ^39^ Zenodo: ConFiRMa dataset_03: simulation of tests on CRM strengthened masonry structures with the OOFEM code (intermediate, multi-layer level modelling). https://doi.org/10.5281/zenodo.7220949 ^30^ This dataset contains the following underlying data: - L01_W (Folder containing the model used for the calibration of GFRP layer parameters) - L04_C (Folder containing the model used for the calibration of the mortar layer parameters) - L07_TS (Folder containing the model for the direct tensile test on CRM coupon) - L09_IS (Folder containing the model for the in-plane shear test on CRM thin slabs) - L10_M (Folder containing the models used for the calibration of the masonry layer parameters) - L11_DC (Folder containing the models for the simulation of diagonal compression tests DC) - L12_IB (Folder containing the models for the simulation of in-plane bending tests IB) - L13_OB (Folder containingthe models for the simulation of out-of-plane bending tests OB) - L14_P (Folder containing the models for the simulations of tests on piers) - L15_S (Folder containing the models for the simulations of tests on spandrels) - L16_GB (Folder containing the models for the simulations of tests on the building) Data are available under the terms of the Creative Commons Attribution 4.0 International license (CC-BY 4.0).
