# Supplementary material for: Multi-layer modelling of masonry structures strengthened through textile-reinforced mortar
Source: Open Res Eur. 2023 Feb 21;2:132. Originally published 2022 Nov 30. [Version 2] doi: 10.12688/openreseurope.15233.2 (PMC10445843; doi:10.12688/openreseurope.15233.2)
Supplement: Peer review history

# Peer review: Reviewer response for version 2

**DOI:** 10.21956/openreseurope.16953.r31977

**Published:** 2023-06-20

**Authors:** Larisa Garcia-Ramonda

**Affiliations:** 1 Universitat Politecnica de Catalunya, Barcelona, Catalonia, Spain

The present work deals with the development of numerical methods for the study on the structural performances of TRM strengthened masonry. The paper focuses on the multi-layer modelling (MLM) approach. The strengthening technique investigated is the composite reinforced mortar (CRM) with GFRP. The numerical simulation is done with an open source code for finite element modelling and the models are composed of 20-node bricks elements. Different scales of different experimental tests have been modelled with a good agreement with their experimental counterpart. From tensile test of CRM coupons, to flexural tests, in plane tests; such as shear compression and diagonal compression tests, and out of plane test on masonry piers, to finally reach the spandrel and building level. The research methodology followed is sound and robust and the conclusions drawn are meaningful to better understand the global behaviour of TRM strengthening solutions. The research overcomes one of the main problems steam from the experimental research, this is a very limited number of test are available for the validation at large scale. The research provides a database for the behaviour estimation of piers and spandrels.

 Next the reviewer presents some issues that may help improve the reading of the document and its reproducibility:

- The layered cross section does take into consideration the the fiber is in between two layers of mortar, but applied directly on top of the masonry. Is the perfect bond condition enough to ensure that the failure modes of the GFRP are correctly captured?
- How was the tensile strength computed from the diagonal compression test? using ASTM/RILEM standards?
- In the building case, the reduction of 80% of the parameters is due to the fact that the specimen was tested and then repair with CRM?

Is the study design appropriate and does the work have academic merit?

Yes

Is the work clearly and accurately presented and does it cite the current literature?

Yes

If applicable, is the statistical analysis and its interpretation appropriate?

Not applicable

Are all the source data underlying the results available to ensure full reproducibility?

Yes

Are the conclusions drawn adequately supported by the results?

Yes

Are sufficient details of methods and analysis provided to allow replication by others?

Yes

Reviewer Expertise:

Experimental testing, masonry, numerical modelling, masonry strengthened with TRM solutions.

I confirm that I have read this submission and believe that I have an appropriate level of expertise to confirm that it is of an acceptable scientific standard.

# Peer review: Reviewer response for version 2

**DOI:** 10.21956/openreseurope.16953.r31975

**Published:** 2023-06-15

**Authors:** Pietro Meriggi, Elisabetta Monaldo

**Affiliations:** 1 Roma Tre University, Rome, Lazio, Italy

**OVERVIEW** 

 The paper describes FEM numerical simulations aiming at validating a novel multi-layer modelling approach for the evaluation of the effects of inorganic-based composites when used for the strengthening of masonry constructions. 

 The validation is carried out through comparison between numerical results, coming by the performance of non-linear static analysis at several scales - elementary specimen, structural element and building, and experimental outcomes derived by other scientific works available in the literature.

 In the proposed multi-layer approach, implemented in the open-source code OOFEM, masonry, strengthened by mortar-based composites, is modelled as a sequence of three layers – masonry, mortar coating and textile, perfectly bonded with each other. The results showed in the work highlight the promising potentialities of the prosed model, which proved suitable for numerical simulations of TRM-strengthened masonry structures under different loading conditions, and could be of notable help for both researchers and practitioners in the field of masonry retrofitting.

 Overall, the paper is very well written, the scope is clear, results are pretty well described and the language is appropriate throughout the text. For these reasons, the reviewers recommend with pleasure its indexing. 

 Some suggestions and minor revisions are listed below, should be useful to enhance the clarity and the completeness of the paper. 

****

**GENERAL COMMENTS**

- In both the introduction and the abstract, it appears that the proposed multi-layer model can be applied to strengthened masonry constructions, irrespective of the type of mortar-based composite used. Nonetheless, after the introduction, the attention is immediately focused on Composite Reinforced Mortar (CRM) systems, and in particular those equipped with GFRP grids, which are notoriously different (in covering layer thicknesses, mesh spacing, application technologies, and consequently also in structural behaviour) from Textile Reinforced Mortar (TRM) or Fabric Reinforced Cementitious Matrix (FRCM) composites. This distribution of paper’s sections might be misleading and not duly highlight the universality of application of the proposed approach, which is undoubtedly one of his strengths. Therefore, the author is suggested to highlight this aspect by explicitly mentioning it in the description of the methodology, which perhaps should be included immediately after the introduction. 
- Throughout the text, explicit reference is made to the software used for the simulations. Notation is chosen accordingly, and reference is made to the OOFEM manual for anything not specified in the text. In order to improve the understanding and usability of the scientific work for researchers interested in the topic but not experts in OOFEM, it is recommended to add clarifications that the author deems necessary. 
- For the sake of clarity, irrespective of the test scale, author is invited to provide a more detailed discussion in the text on the failure modes numerically experienced, supported by quantitative measures. 

**SPECIFIC COMMENTS**

 Keywords: acronyms might be introduced by their meaning. 

 Section “Main features and calibration of the numerical model” 

- Page 5: In the description of the modelling of the CRM, it would be clearly stated that the material is considered not resistant in compression, as shown in Table1; 
- Table 1: Author should explain what “OOFEM equivalent strain type” stands for; 

- Table 1: at the bottom of the table, the author refers to default values for unspecified parameters. Perhaps, for the sake of completeness, these parameters could be introduced and briefly described. Accordingly, the author should add similar clarifications in Tables 2 and 3; 
- Table 2: the meaning of all the symbols introduced here should be described in the table itself or in the text. If the parameter f_t1 refers to a strength, the unit of measure may be indicated. Tensile and compressive strengths are reported with the same symbol (fc); please correct it. 
- Figure 4a: Author should report in the legend that the grey area indicates the experimental result;
- Figure 5a: Author should also plot experimental results; 
- Table 3: Tensile and compressive strengths are reported with the same symbol (fc). The values of some strengths are given without units (see B2 and B1). For the sake of clarity, for each type of masonry indicated in the table, it might be useful to add the corresponding reference. As regards the softening law, please specify if it refers either to tension or compression.

- Page 7: Author should add references to of the “available tests on plain masonry samples”. 

Section “Elementary specimen level” 

- For the sake of clarity, author could add a figure with a schematic representation of the set-up of the three experimental tests (as in [28])
- In the discussion of results, a comparison between experimental and numerical failure modes could be added; 

- Author is invited to choose between Young's modulus and Young modulus and edit the text accordingly. Furthermore, Poisson modulus might be Poisson ratio. 
- Figure 6: Author is invited to specify to which simulation the three different plots refer (i.e. type of masonry for (a), axial stress level for (b)). This figure shows the distribution of a principal strain. Please specify which one by providing a reference system for each configuration. The same comment is also valid for Figures 13, 17 and 20. 
- Figure 7: Numerical curves cannot be well distinguished from experimental ones, due to the use of the same colour (even if the line thickness changes). The same comment is also valid for Figures 8, 9 and 11. Furthermore, in the caption, author should provide a brief description of sub-figures a, b and c. The same comment is also valid for Figures 8, 11, 13, 15, 17 and 20.

Section “Structural element level” 

- Sub-section “Masonry piers”: in the second paragraph author states that “[…], a perfect bond was assumed between the masonry pier and the RC beam”. A justification of this assumption should be provided, by explaining how this condition was achieved in the experimental tests; 

Is the study design appropriate and does the work have academic merit?

Yes

Is the work clearly and accurately presented and does it cite the current literature?

Yes

If applicable, is the statistical analysis and its interpretation appropriate?

Not applicable

Are all the source data underlying the results available to ensure full reproducibility?

Yes

Are the conclusions drawn adequately supported by the results?

Yes

Are sufficient details of methods and analysis provided to allow replication by others?

Partly

Reviewer Expertise:

Pietro Meriggi: Seismic engineering, Masonry structures, Externally bonded strengthening materials, Experimental investigations, Distinct Element modelling (DEM), Analytical modelling; Elisabetta Monaldo: Mechanics of composite materials, Computational modelling, Finite element method.

We confirm that we have read this submission and believe that we have an appropriate level of expertise to confirm that it is of an acceptable scientific standard.

# Peer review: Reviewer response for version 1

**DOI:** 10.21956/openreseurope.16475.r30539

**Published:** 2023-01-30

**Authors:** Bartolomeo Pantò

**Affiliations:** 1 Department of Engineering, Durham University, Durham, UK

The paper presents a simplified macro-scale modelling approach to describe the nonlinear response of masonry walls reinforced by fibre-reinforced layers applied on the external masonry surfaces. The model is validated by the comparison with experimental tests and the predictions of microscale FEM simulations. The paper is well-written and organised, providing a comprehensive description of the nonlinear behaviour of different masonry typologies reinforced by fibre-reinforced materials. Moreover, the topic interests researchers and engineers in assessing and retrofitting historical and cultural heritage assets. For these reasons, the paper is worth publishing. Some minor comments and proposals for changes are reported below to help the author to improve the paper further.

 Specific comments:

- Abstract: in the “Result” section, reinforced masonry structures might be mentioned in addition to existing masonry.
- Introduction
  - (second line): refurbishment interventions might be retrofitting interventions.
  - Fourth-last lines of the second paragraph: materials combinations might be material combinations.
- “Main features and calibration of the numerical model” section:
  - How was the mesh size (167x167mm) determined? Was the model sensitivity on the mesh determined? Maybe the author can refer to previous work by including a citation.
  - How many integration gauss points are used?
  - What are the “plaster parameters” mentioned on page 5? Does it refer to the reinforcement mortar layer?
  - What are the “Equivalent tensile strains” mentioned on page 6?
  - Page 22, last paragraph: citation to ref 22 and 34 should be formatted as subscripts.
- Figure 4b: “experimental” might be “experiment”
- Page 7: the sentence “experiment-numerical comparison” might be revised.
- Table 3, please explain how have the material parameters been evaluated.
- Figure 6: explains in the text the meaning of the colormap. It should be clarified (in this figure and in the following ones, what layer the shown strains are relative to since the strains should be variable along with the thickness.    
- Figure 7: describe how has the tensile strain been evaluated.
- Page 10: “Structural Element Level”: additional information should be provided about experimental tests. Have they already been published? If yes, the reference should be included. The author is referring to the parameters in Table 3. Have these masonry typology used in the experimental campaign? Or only in the simulations?  
- Figure 10b: the text is unclear.
- Page 12, first paragraph: what is the additional axial load applied to the panel? Again, it is unclear why the parameters in Table 3 are considered here. The sentence “…by performing direct linear interpolation within the range, starting from the calculated tensile strength”. What are the softening parameters that guarantee the best fit of experiments? Please, include a reference to laboratory tests.
- Page 12: The sentence “For the Young modulus, the initial values (till 15% of the compressive strength) were set equal to 3 times the values estimated from 41, to correctly account for the initial higher stiffness and the progressive stiffness degradation” is unclear. Please, revise it.
- Figure 13: the displacement magnitude of numeric predictions and tests should be reported in the figure or in the text. For each mechanism, 2 images of the real mechanism are shown. What does each one refer to?   
- Building level: “results of recently performed”. Please, add a reference. What does “id GB” mean?
- How have wooden floors been modelled?
- Page 16: what does “plaster discontinuity” mean?
- Page 16: the sentence “grid (layer equivalent properties: elastic-plastic behavior with Young modulus E = 20.8 GPa,” should be improved. What does “elasto-palstic” mean? Isn’t 15.8% a very high ultimate deformation limit? Young modulus might be Young’s modulus.   
- Figure 20: figures from tests reported in the right part appear different from those reported in the left part of the figure. Why? The displacement level of each shown model and experiment might be declared in the figure or the text.

Is the study design appropriate and does the work have academic merit?

Yes

Is the work clearly and accurately presented and does it cite the current literature?

Yes

If applicable, is the statistical analysis and its interpretation appropriate?

Yes

Are all the source data underlying the results available to ensure full reproducibility?

Yes

Are the conclusions drawn adequately supported by the results?

Yes

Are sufficient details of methods and analysis provided to allow replication by others?

Yes

Reviewer Expertise:

numerical models for nonlinear analyses, structural analysis, and seismic assessments.

I confirm that I have read this submission and believe that I have an appropriate level of expertise to confirm that it is of an acceptable scientific standard.
